# Cooperation and opportunism in Galapagos sea lion hunting for shoaling fish

**DOI:** 10.1002/ece3.7807

**Published:** 2021-06-21

**Authors:** Tui De Roy, Eduardo R. Espinoza, Fritz Trillmich

**Affiliations:** ^1^ The Roving Tortoise Worldwide Nature Photography Golden Bay New Zealand; ^2^ Galápagos National Park Directorate Isla Santa Cruz, Galápagos Ecuador; ^3^ Instituto Nacional de Biodiversidad (INABIO) Quito Ecuador; ^4^ MigraMar Olema CA USA; ^5^ Department of Animal Behaviour University of Bielefeld Bielefeld Germany

**Keywords:** collaboration, coordination, scad (*Decapterus muroadsi*), sociality, *Zalophus wollebaeki*

## Abstract

For predators, cooperation can decrease the cost of hunting and potentially augment the benefits. It can also make prey accessible that a single predator could not catch. The degree of cooperation varies substantially and may range from common attraction to a productive food source to true cooperation involving communication and complementary action by the individuals involved. We here describe cooperative hunting of Galapagos sea lions (*Zalophus wollebaeki*) for Amberstripe scad (*Decapterus muroadsi*), a schooling, fast swimming semipelagic fish. A group of 6–10 sea lions, usually females only, drove scad over at least 600–800 m from open water into a cove where, in successful hunts, they drove them ashore. Frequently, these “core hunters” were joined toward the final stages of the hunt by another set of opportunistic sea lions from a local colony at that beach. The “core hunters” did not belong to that colony and apparently were together coming toward the area specifically for the scad hunt. Based on the observation of 40 such hunts from 2016 to 2020, it became evident that the females performed complementary actions in driving the scad toward the cove. No specialization of roles in the hunt was observed. All “core hunters” and also opportunistically joining sea lions from the cove shared the scad by randomly picking up a few of the 25–300 (mean 100) stranded fish as did scrounging brown pelicans. In one of these hunts, four individual sea lions were observed to consume 7–8 fish each in 25 s. We conclude that the core hunters must communicate about a goal that is not present to achieve joint hunting but presently cannot say how they do so. This is a surprising achievement for a species that usually hunts singly and in which joint hunting plays no known role in the evolution of its sociality.

## Graphical abstract

We describe cooperative hunting of groups of Galapagos sea lions (*Zalophus wollebaeki*) for Amberstripe scad (*Decapterus muroadsi*), a schooling, fast swimming fish which they drive ashore by complimentary actions. The “core hunters” were together coming to the area specifically for the scad hunt. We conclude that the core hunters must communicate about a goal that is not present to coordinate joint hunting which is a surprising achievement for a species that usually hunts singly and in which joint hunting plays no role in the evolution of its sociality.

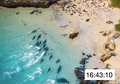

## 1 INTRODUCTION

Hunting is an energy‐intensive, potentially dangerous activity for a predator, particularly when the prey is large and when hunting exposes the predator itself to other predators. The obvious benefit for the predator is energy intake that covers the cost of living and potentially of reproduction. On the contrary, the prey is under strong selection to successfully evade predators. This arms race between predator and prey makes hunting potentially very costly. Cooperation represents one way to increase hunting success, reduce the cost of hunting and its dangers, and potentially increase benefits. Foraging benefits are only accrued if communal hunting increases the rate at which prey is caught, or the total amount of prey obtained, so the shared rewards increases per capita net benefit (Creel, 2001). In addition, the direct benefits and costs of cooperative hunting may depend on prey size relative to the predator, while the indirect ones such as inclusive fitness may depend on social and kin relationships among the hunters. Prey size and grouping tendency determine the extent to which hunters can share the resource and how intense competition may become, if the hunt is successful.

The level of behavioral organization between co‐operators varies substantially, and cheating may emerge as an alternative strategy to reduce the costs to self while participating in the benefits of a successful hunt (Packer & Ruttan, 1988). This complicates the description of communal hunting behavior. At the simplest level, there is (1) mere similarity of action between individuals that happen to hunt in spatial proximity; (2) acts may also be performed in synchrony (i.e., similar behavior shown in unison); there may be (3) coordination (similar acts performed at the same place and time); and finally, (4) true collaboration (complementary acts performed at the same place and time). Recently, Lang and Farine (2017) pointed out that additionally cooperative hunting may usefully be characterized multidimensionally by the degree of sociality, communication, specialization within the hunting group, the extent of resource sharing and dependence, that is, the importance of social predation for the overall energy intake of the individual.

Most early work on cooperative hunting has been done on terrestrial species. In some species, such as hunting dogs (*Lycaon pictus*), hunting together most likely has selected for their extreme sociality (Creel, 2001). For them, being able to defend the prey against stronger competitors may additionally select for the evolution of group hunting. In others, like hyena (*Crocuta crocuta*) and lions (*Panthera leo*), communal hunting is frequently observed but whether it has been the prime selective force is disputed (Caro, 1994; Creel, 2001; MacDonald, 1983; Packer & Caro, 1997). Hyenas jointly defend territories that provide food resources for the clan (Kruuk, 1972; Tilson & Hamilton, 1984). In lions, communal care and protection of offspring may be just as important in selecting for female sociality as communal hunting (MacDonald, 1983). This may also apply for sperm whales (*Physeter macrocephalus*; Whitehead & Weilgart, 2000).

In the marine environment, cooperative hunting has been documented for fish (Bshary et al., 2006; Johnson & Chase, 1982), dolphins (Benoit‐Bird & Au, 2009; Connor, 2000; interspecifically: Daura‐Jorge et al., 2012; Vaughn et al., 2007), and whales, especially for humpback and killer whale (*Megaptera novaeangliae*; Clapham,  *Orcinus* *orca*; Baird, 2000; Similä & Ugarte, 2012). There is much less evidence for cooperation in pinnipeds, even though their cognitive capacity matches that of terrestrial predators (Würsig 2018) and certainly that of fish.

In pinnipeds, grouping clearly evolved through other mechanisms than in terrestrial predators (avoidance of predators of newborns, reduction in harassment of adult females by males, etc.) (Bartholomew, 1971; Trillmich & Trillmich, 1984). Cooperative hunting can be excluded as an important selective force for their sociality. When many pinnipeds hunt at a common site, this is usually caused by independent attraction to an important food resource like migrating salmon at river mouths for California sea lions (*Zalophus californianus*; Keefer, Stansell, Tackley, Nagy, Gibbons et al., 2012). Recently, cooperative hunting has been reported for Galapagos sea lions (*Zalophus wollebaeki*) attacking large yellow‐fin tuna (*Tunnus albacares*) on the northern coast of the island of Isabela, Galapagos (Páez‐Rosas et al., 2020).

We here report another hunting strategy by Galapagos sea lions directed at schooling prey, in which a coordinated group of sea lions herd their prey through open water toward a predetermined stranding site. In particular, we ask what degree of cooperation this collaborative behavior involves.

## 2 METHODS

T.D.R. observed the sea lions hunting for Amberstripe scad (*Decapterus muroadsi, Temminck* *& Schlegel 1843*) at Rocas Bainbridge (90°33′52″W,0°21′00″S), a group of 6 islets directly east of the island of Santiago in the Galapagos archipelago. This scad species reaches 55cm in length and is a semipelagic planktivore feeding in dense schools primarily on fish eggs and larvae.

**FIGURE 1:**
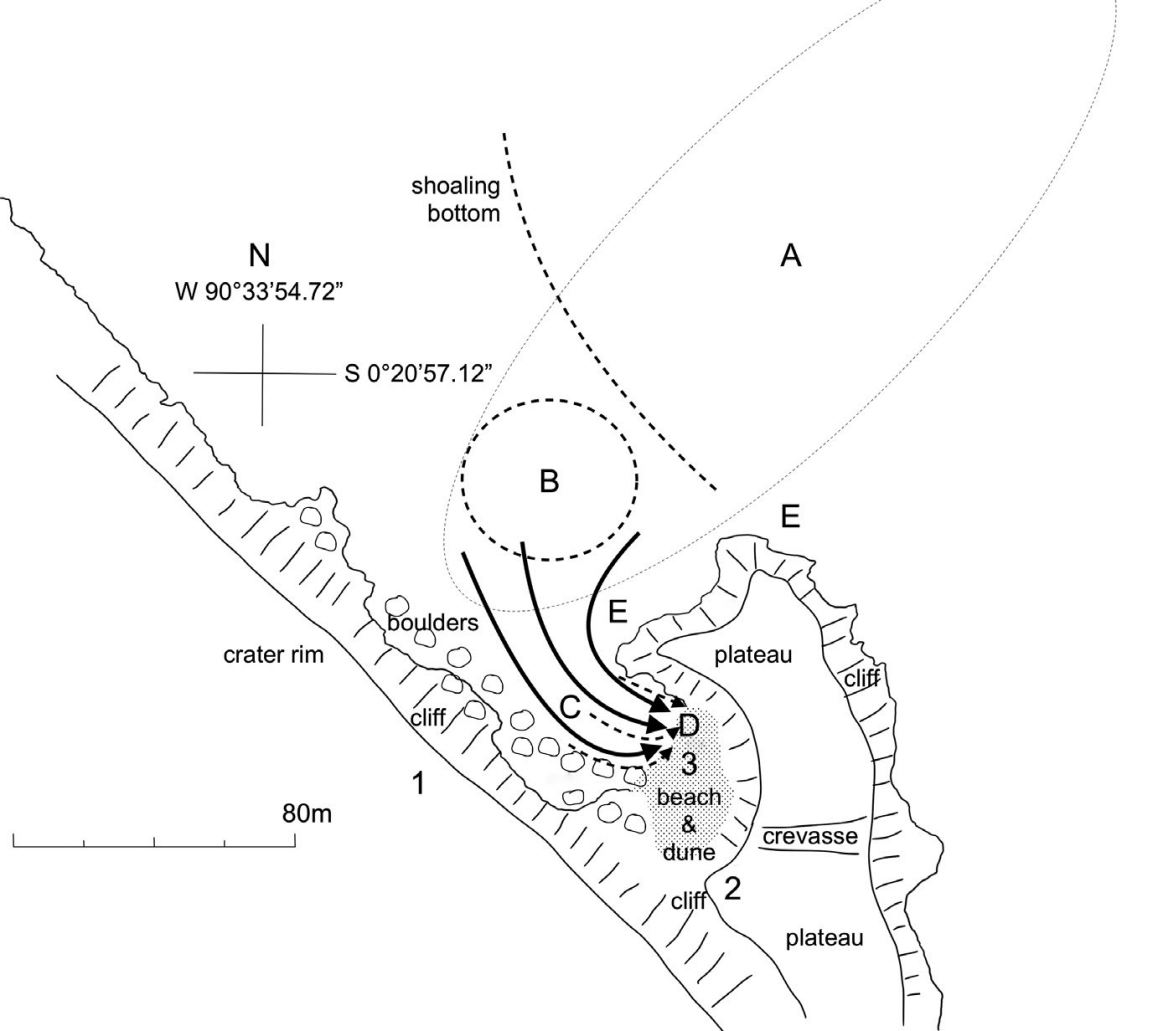
Map of Bainbridge rock where the hunts ended. Numbers refer to observation points (1) on cliff edge, 35 m above sea level (asl), (2) at top of dune and lower cliff edge, 10m asl, (3) on beach, at sea level. (a) General area where opportunistic hunters from the local colony met incoming core hunters (dotted line). (b) Area where almost all hunts faltered (dotted circle), sometimes for several minutes, with sea lions porpoising in various directions before regrouping for the final push to shore. (c) Pathways of sea lions swimming at top speed on final drive to stranding (solid lines) and fish when these became visible (dotted lines). (d) Most frequent stranding area near intersection of beach and rocks. (e) Areas off of both rocky points where opportunistic hunters from the local colony, up to eight females and one bull in the most recent hunts, were often swimming around or floating, but raising their heads high and looking out to sea whenever pelicans flew over. Graphic by Mark Jones

Data are based on 284 hr of observation over 31 days, between 3 September 2016 and 13 November 2020, during which time 40 hunts were observed (Table 1). Observations were made six times from shore level, seven times from about 3 m high, 13 times from a height of about 10 m, and 14 times from a vantage point about 35 m above the beach which allowed a view of the adjacent sea for more than a kilometer out (Figure 1, point 1). During this time, much of the events were documented by photography which allowed to estimate the number of fish chased ashore as well as to record the duration of the fast‐paced feeding behavior from the time stamps of the photographs. By analyzing the resulting 2,400, time‐stamped photographs in detail, a considerable amount of information was extracted that could otherwise not have been recorded accurately via observation alone. For example, in 22 of the 32 successful hunts recorded, the number of fish driven ashore by the sea lions was estimated to the nearest 25, ranging from under 25 to ~300.

**TABLE 1:** Detailed data on the 40 observed hunts

Date	Hunt no.	daytime	Duration of observation (hr)	Time of hunt	Observation site	Approx altitude (m)	Active hunters	locals meeting hunters	Total sea lions	Hunting success	Estimated # fish stranded (to nearest 25)	Time spent feeding (seconds)	Pelicans & other scavengers	Photos	Stranding location
03.09.2016	N/A	17:17–17:27	0,17		Clifftop	35	N/A	N/A	N/A	No hunt	N/A	N/A	No data	N	N/A
04.09.2016	N/A	6:17–8:11			Beach	0	N/A	N/A	N/A	No hunt	N/A	N/A	No data	N	N/A
04.09.2016	**1**	10:22–11:36	1,90	15:23	Clifftop	35	10+	4	16	Yes	<25	11	7	Y	beach
04.09.2016	**2**	15:15–16:51	1,60	16:43	Clifftop	35	8+	No data	19	Yes	150	18	6	Y	beach
27.10.2016	**3**	8:30–18:30	10,00	12:18	Dune	10	No data	No data	7++	Yes	No data	No data	5	Y	beach
27.10.2016	**4**			12:22	Dune	10	No data	1+	12+	Yes	No data	5	6	Y	rocks
27.10.2016	**5**			12:40	Dune	10	6+	2+	11+	Yes	150	18	6	Y	beach
27.10.2016	**6**			12:46	Dune	10	7+	3+	12	Yes	50	45	8	Y	rocks
27.10.2016	**7**			13:19	Rocky shore	3	No data	No data	13	Yes	No data	9	4	Y	rocks
27.10.2016	**8**			14:01	Rocky shore	3	No data	No data	9++	Yes	25	34	5	Y	rocks
27.10.2016	**9**			14:07	Rocky shore	3	No data	No data	7++	Yes	>25	7	6	Y	rocks
27.10.2016	**10**			14:31	Rocky shore	3	No data	No data	6++	No	N/A	N/A	N/A	N	N/A
27.10.2016	**11**			15:00	Rocky shore	3	5++	3	15	Yes	No data	2	8+	Y	beach
27.10.2016	**12**			15:34	Dune	10	No data	No data	3+++	Yes	No data	No data	5+	Y	rocks
27.10.2016	**13**			16:02	Rocky shore	3	No data	No data	14	Yes	150	58	6+	Y	beach
27.10.2016	**14**			16:21	Beach	0	No data	No data	8++	Yes	25	19	3+	Y	beach
27.10.2016	**15**			16:30	Beach	0	No data	No data	12+	Yes	200	62	6	Y	beach
28.10.2016	N/A	6:00–14:00	8,00		Dune	10	N/A	N/A	N/A	No hunt	N/A	N/A	Present	N	N/A
15.06.2017	N/A	8:00–18:30	10,50		Dune	10	N/A	N/A	N/A	No hunt	N/A	N/A	Present	Y	N/A
16.06.2017	N/A	6:00–16:00	10,00		Dune	10	N/A	N/A	N/A	No hunt	N/A	N/A	Present	Y	N/A
08.07.2020	**16**	8:00–18:30	10,50	16:12	Dune	10	6	4	10	Yes	25	12	2	Y	beach
08.07.2020	**17**			16:43	Dune	10	5	3	8	Yes	75	16	3	Y	beach
08.07.2020	**18**			16:53	Dune	10	No data	No data	Yes	Yes	No data	No data	Present	N	beach
08.07.2020	**19**			18:30	Dune	10	No data	No data	Yes	Yes	No data	No data	Present	N	rocks
09.07.2020	N/A	6:00–18:30	12,50		Dune	10	N/A	N/A	N/A	No hunt	N/A	N/A	Present	N	N/A
10.07.2020	N/A	6:00–18:30	12,50		Dune	10	N/A	N/A	N/A	No hunt	N/A	N/A	Present	N	N/A
11.07.2020	N/A	06:00–8:00	2,00		Dune	10	N/A	N/A	N/A	No hunt	N/A	N/A	Present	N	N/A
24.10.2020	**20**	13:30–17:00	3,50	14:40	Beach	0	No data	No data	9+	Yes	100	91	10++	Y	beach
24.10.2020	**21**			15:00	boat	0	No data	No data	No data	unknown	No data	No data	No data	N	N/A
24.10.2020	**22**			15:15	South coast	3	No data	No data	No data	unknown	No data	No data	No data	N	unknown
25.10.2020	**23**	6:20–17:30	11,17	07:35	Clifftop	35	6	No data	6+	Yes	25	42	26 + 2 booby	Y	beach
25.10.2020	**24**			08:02	Clifftop	35	6	8++	21	Yes	75	12	26 + 1 booby	Y	beach
25.10.2020	**25**			08:25	Clifftop	35	6	6	12	Yes	25	36	20 + 1 booby	Y	beach
25.10.2020	**26**			10:33	Clifftop	35	6	6+	12+	Yes	50	20	27	Y	beach &+ rocks
25.10.2020	**27**			11:14	Clifftop	35	6	8	14	Yes	150	21	26 + 1 booby, 3 sharks	Y	beach & rocks
25.10.2020	**28**			12:43	Clifftop	35	6	6+	16	Yes	75	13	27 + 2 booby, 1 sharks	Y+video	beach
26.10.2020	N/A	6:45–17:00	10,25		Clifftop	35	N/A	N/A	N/A	No hunt	N/A	N/A	Present	N	N/A
27.10.2020	N/A	7:00–17:15	10,25		Clifftop	35	N/A	N/A	N/A	No hunt	N/A	N/A	Present	N	N/A
28.10.2020	N/A	06:45–17:10	10,42		Clifftop	35	N/A	N/A	N/A	No hunt	N/A	N/A	Present	N	N/A
29.10.2020	N/A	06:45–16:40	9,92		Clifftop	35	N/A	N/A	N/A	No hunt	N/A	N/A	Present	Y	N/A
30.10.2020	**29**	06:50–17:50	11,00	14:10	Clifftop	35	6	4	10	No	N/A	N/A	Present	Y	N/A
31.10.2020	**30**	06:00–17:00	11,00	07:34	Clifftop	35	No data	No data	12	No	N/A	N/A	8+	Y	N/A
01.11.2020	N/A	05:50–16:30	10,67		Clifftop	35	N/A	N/A	N/A	No hunt	N/A	N/A	Present	N	N/A
02.11.2020	**31**	06:50–17:40	10,83	15:15	Clifftop	35	6	4	10	Yes	300	36	4	Y	beach
03.11.2020	N/A	6:50–17:00	10,17		Dune	10	N/A	N/A	N/A	No hunt	N/A	N/A	Present	N	N/A
04.11.2020	N/A	6:50–17:00	10,17		Clifftop	35	N/A	N/A	N/A	No hunt	N/A	N/A	Present	N	N/A
05.11.2020	N/A	6:50–17:00	10,17		Clifftop	35	N/A	N/A	N/A	No hunt	N/A	N/A	Present	N	N/A
06.11.2020	**32**	7:00–16:30	9,50	08:45	Clifftop	35			8	No	N/A	N/A	17 + 4 boobies	Y	N/A
07.11.2020	**33**	7:00–16:00	9,00	07:09	Clifftop	35	6	4	10	Yes	50	10	11+	Y	beach
08.11.2020	N/A	7:00–16:30	9,50		Clifftop	35	N/A	N/A	N/A	No hunt	N/A	N/A	Present	N	N/A
09.11.2020	N/A	7:00–16:30	9,50		Clifftop	35	N/A	N/A	N/A	No hunt	N/A	N/A	Present	N	N/A
10.11.2020	N/A	7:00–16:30	9,50		Clifftop	35	N/A	N/A	N/A	No hunt	N/A	N/A	Present	N	N/A
11.11.2020	N/A	7:00–16:00	9,00		Dune	10	N/A	N/A	N/A	No hunt	N/A	N/A	Present	N	N/A
12.11.2020	N/A	7:00–16:00	9,00		Clifftop	35	N/A	N/A	N/A	No hunt	N/A	N/A	Present	N	N/A
13.11.2020	**34**	7:00–17:00	10,00	11:09	Beach	0	No data	No data	8++	Yes	No data	5	28	Y	beach
13.11.2020	**35**			11:43	Beach	0	No data	No data	8++	Yes	No data	24	26	Y	beach
13.11.2020	**36**			12:13	Dune	10	No data	No data	7++	Yes	150	26	26	Y	beach
13.11.2020	**37**			13:42	Dune	10	6	0	6	No	N/A	N/A	0	Y	N/A
13.11.2020	**38**			14:53	Dune	10	6	2+	8+	Yes	No data	4	13+	Y	rocks
13.11.2020	**39**			15:40	Dune	10	6	3	9	Yes	100	28	22+	Y	beach
13.11.2020	**40**			16:02	Dune	10	6	No data	No data	No	N/A	N/A	Present	Y	N/A

Notes

In cases when only a few sea lions were detectable (in the photographs) but the observer was sure that more were present, we note this by adding a “+” to the number, meaning that there were at least a few more than what could be counted. When ++ is used, this means that there were many more than counted, but no estimate was possible.

Generally, as hunting sea lions porpoise intensely to maintain high speed, it is impossible to determine accurately the exact number of individuals involved, because they do not surface in unison. In addition, the observer was taking photographs to document events and, when using a telephoto lens (zoom range 80–400 mm), was not always able to observe the whole scene. This limited the accuracy of the counts of core hunting animals versus local sea lions joining the hunt opportunistically closer to shore, usually within 100–200 m of the site where fish were driven ashore. When hunts were observed from sea level, this angle of view allowed only to state that a hunt occurred but not to detail the number or role of individuals involved. As a consequence of these limitations, we here report the minimum number of sea lions observed, whether considered core hunters (those that drove the fish school toward the island in a zigzag course sometimes exceeding 1,000 m distance) or the opportunistic individuals that joined the hunt near its termination. Because it was very difficult, and often impossible, to distinguish the “core hunters” from the “opportunistic hunters” (from the local beach) who joined the hunt in the final minutes, the total number of sea lions observed is not necessarily the sum of the two categories (Table 1, which gives the details for all 40 hunts). Usually, brown pelicans (*Pelecanus occidentalis*) joined the hunt and their numbers were also estimated unless they were highly dispersed or the hunt failed before the sea lions came close to the beach.

In the final seconds of the hunt when the sea lions appeared to accelerate to maximum speed, their swim speed was estimated to be around 4.5 m/s from the time stamp of photographs and the distance estimate derived from Google Earth. This value lies well within the range of swim velocities (usually around 2 m/sec, max 5.3 m/s) reported by Ponganis et al. (1990). For Atlantic mackerel (*Scomber scombrus*) of about 30–40 cm length, a species comparable to Amberstripe scad, a sustained speed of 1.2 m/s (Wardle et al., 1996) and a maximum burst speed of 5.5 m/s have been reported (Wardle & He, 1988).

## 3 RESULTS

During 31 days (284‐hr observation time), hunts were observed on only 11 days (data in Table 1). On five of these days, only one hunt was observed, and maximally, 13 hunts occurred within one day (median 1.5 hunts/day). Only six out of 40 hunts failed and for two the outcome was unknown as the cove could not be seen from the observer's position. The observation effort was equally distributed across daytime, but 30 out of 40 hunts happened during the afternoon (12:00–18:30 hours). In two cases, the hunts’ duration was timed when the hunters were still 600m and 800m from shore, respectively. The hunts lasted between five and six min until they ended at the shore. Of course, the length of the zigzag course followed by the hunters was greater than the linear distance of visibility. The time sea lions spent feeding once the fish had beached was very short, lasting on average 24 ± 20 s (mean and *SD*; *n* = 28 hunts; range 2 to 91 s). In 2020, six sea lions were estimated to be the core hunters (except one case of five). In 2016, in the five cases where the number of hunters could be estimated more than seven sea lions were involved (Table 1).

As the core hunters approached the shore toward the end of the hunt, they were joined by sea lions from the cove adding up to a mean of 12.4 total sea lions estimated at the stranding site. In successful hunts, a mean of about 100 fish stranded (range 25–300, estimated in sets of 25).

### 3.1 Description of a hunt

About eight hunts could be observed from the beginning to the end, or at least from the farthest point (500–800 m) that visibility allowed. They most often appeared from an easterly direction skirting around the outermost of the Bainbridge Rocks. Often, it was the flight of pelicans (usually ranging between four and eight) that alerted the observer to the hunt, before the sea lions were detected.

**FIGURE 2:**
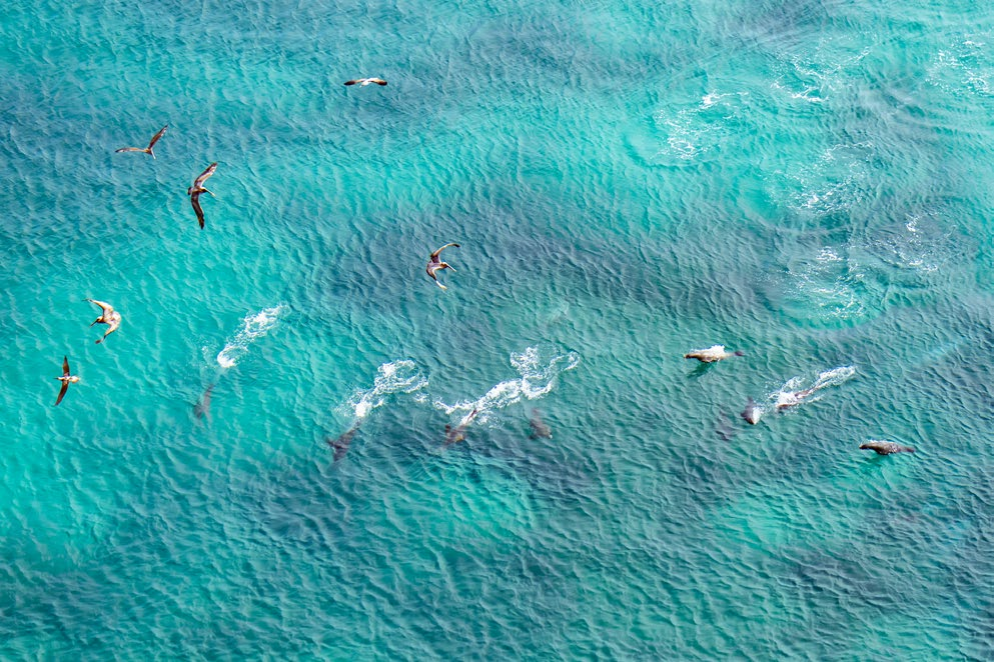
As the school is being driven into shallow water (point B in Figure 1), the hunters fan out more widely, dive deep, and criss‐cross to direct the fish toward the cove, while pelicans wheel overhead

Under good conditions, one could detect several sea lions porpoising just beyond the island, almost always on its northern side. They porpoised nonstop, coming fast toward the small cove just below the observation point. Average speed in this approach (roughly 800 m in hunt #38) was estimated at 2.2 m/s. Sometimes, they came in a fairly straight line, but more often swam in a widely zigzagging course, often detouring in a southerly direction toward the open ocean before doubling back toward their target. The changes of direction and deeper dives were usually undertaken more or less simultaneously by all or most animals. At times, the entire group disappeared together while chasing the fish southward, then reappeared a minute or two later heading once again to the cove. In good visibility, six large female sea lions (recognizable by their sleek shape and size) were porpoising parallel to each other with a separation distance between individuals of 2–15 m (Figure 2a). As they approached the north point of the cove, sometimes they pressed near the rocky shore, but on other occasions stayed between 50 and 100m from shore, heading toward the mouth of the cove. Here, the seafloor becomes shallower and begins to be visible, with large boulders. Around this area, approximately 100 to 200m from the target destination (Figure 1, point B), there was almost always several minutes, when all sea lions were diving at high speed in different directions (Figure 2), usually fanning out and diving too deep for the observer to maintain visual contact, porpoising fast and erratically when surfacing. Even though the fish were not yet visible to the observer, this may indicate a time when the school was making an effort to escape and the hunters were attempting to round up the fish, snatching very quick breaths of air, and redirect them toward the cove.

In this general area, a number of sea lions from the local colony, including females, bull(s), subadults, and yearlings, would join the chase, swimming out fast to meet the incoming hunters, possibly adding confusion to the hunt. These local, opportunistically joining sea lions often kept an active lookout for circling pelicans, in which they raise the head much more frequently than a resting sea lion would do. On many occasions, seeing circling pelicans induced them to swim out to meet the core hunters, as there was no clear view of the approaching hunt from within the cove. Large numbers of pelicans gathered quickly at this stage, especially if the approach had been visible for a longer time. As the hunt approached the shore, the pelicans circled low over the water just ahead of the sea lions casting dark shadows on the scad school. On days when multiple hunts occurred in fairly rapid succession (Table 1), these local sea lions, ranging from one to nine, remained in near shore waters on the seaward side of the rocky point enclosing the cove. They raised their heads high every few minutes to look toward the open sea and swam farther out as soon as pelicans flew in that direction. Especially, the older local bull, recognizable by his gray pelt, was sometimes seen porpoising with the actively hunting females from several hundred meters out at sea.

**FIGURE 3:**
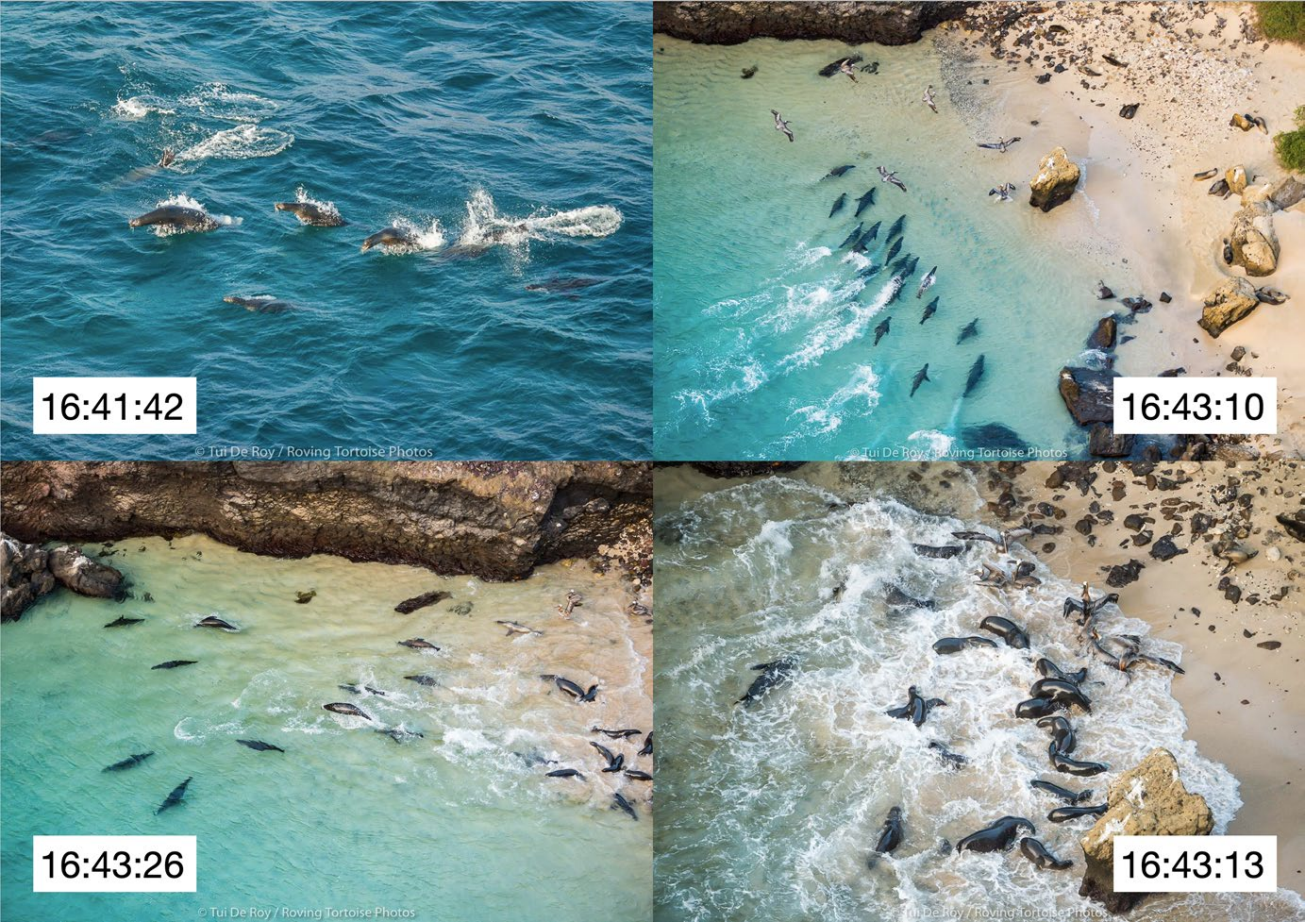
Clockwise from top left: hunting sequence taken from Hunt #2 (Table 1), with time stamps. 16:41:42 Core group of sea lions chase the scad in open water (labeled A on map Figure 1). 16:43:10 Scad school in the shallows being driven onto the beach by the original hunters plus opportunistic hunters that have joined in. 16:43:13 Sea lions and pelicans feeding at the shoreline. 16:43:26 Immediately after feeding, the hunters leave the beach (latter three photographs are of beach area labeled D on map Figure 1)

Once the hunt approached the main shore of the island (large boulders at the base of the tall cliff), all sea lions swam toward the cove in a final chase aimed at the beach, although often a few young individuals darted about erratically. From time‐stamped photographs of hunt #27 (Table 1), this final approach speed was measured to be 4.5 m/s. Invariably, the fish appeared to swim into this trap in a counter‐clockwise fashion. They first followed the boulder shore heading in an easterly direction, then turned sharply northward upon approaching the white sand bottom in shallower water (Figure 1). Only at this stage did the dark green scad school become visible to the human eye. They aimed for the dark, shadowy lava cliff enclosing the cove on its northeast side. The sea lions apparently anticipated this, cutting them off in a pincer movement (Figure 3) and, in the most successful hunts, drove large numbers either directly up onto the sand or, at high tide, behind a large boulder where there was no escape. During this final, all‐out rush, the sea lions often swam in a line with 4–10 animals closely abreast of each other, not breaking the surface but apparently many of them blowing streams of bubbles, possibly as an additional means of scaring the fish onto the beach. The arrival of the sea lions only a few meters behind the fish school created a large enough wave to wash many fish several meters beyond the normal waterline. The sea lions searched frantically for those fish still in a few centimeters of water, jumping over each other to grab the fish (Figure 3), around 40cm long, which they swallowed whole in only a few seconds. In hunt #31, four sea lion females were observed to consume 7–8 fish each in 25 s.

From the moment of stranding, all fish were typically consumed within well under one minute, even when several dozen fish had stranded. In only two of all 28 hunts when feeding times were clocked, was one minute surpassed. In these cases, feeding lasted for 62 and 91 s, respectively, as a result of the fish school being split into two parts, stranding at different points of the beach. Some fish escaped during the final drive into the cove.

Pelicans are very attentive to these hunts. By positioning themselves along the water's edge ahead of the arriving hunt, pelicans could take an estimated 20%–50% of the catch, especially when fish numbers were low.

### 3.2 Changes in hunt dynamics observed over four years

A total of 15 successful hunts were observed during 2016 and 2017, with as many as 19 sea lions involved, displaying high synchrony and strategic positioning in the final stages of the hunt as all animals drove the fish school toward the beach (Figure 3). At that time, the majority appeared to be adult females with a very small number (undetermined) of subadult bulls. Although these hunts were not observed from beginning to end, there was a strong impression that between seven and ten core hunters worked together in close synchrony, versus a smaller number of local opportunistic hunters joining in at the last moment, typically no more than four individuals.

From 25 hunts observed in July and October–November of 2020, it was possible to see the hunt in greater detail. The core group was now reduced to six females, compared with an estimated seven to ten individuals in previous years. The average number of core and opportunistic animals involved was reduced to around 10–14 animals (with two exceptions of 16 and 21, including some yearlings and one or two bulls). These 2020 hunts also appeared less coordinated than in previous years.

## 4 DISCUSSION

We document an example of apparently cooperative hunting by Galapagos sea lions who drive a school of fish into a cove that operates as a trap. Whereas in terrestrial mammals hunting cooperatively will allow them to capture larger prey than a single individual could bring down, in marine mammals cooperative hunting often enables predation on schooling prey where a single predator may hunt very inefficiently. Such hunting cooperation has been described for killer whales hunting herring (Baird, 2000), for humpback whales (Clapham, ), for dolphins (Connor, 2000; Vaughn et al., 2007), and for California sea lions (Pierotti, 1988).

The evidence for collaboration among the core hunters comes from observations of 40 hunts over a period of five years. During all hunts, this core group, usually numbering a minimum of six individuals, worked in synchrony in a manner that involved both (a) consistently driving prey toward a targeted location and (b) close cooperation by porpoising in a well‐defined geometry over a zigzagging course for up to about 1,000 m. The sea lions dove and fanned out simultaneously, apparently controlling the prey school's swim direction. This core group consisted of adult females working together from where the hunt first became visible at the outermost Bainbridge Rock, all the way to the stranding point. The core group did not belong to the local colony and left the area immediately after the conclusion of each hunt, with some local animals following behind them. In contrast to normal, individual foraging, these females must have grouped specifically for this hunt.

In the hunt of scad, the sea lions’ cooperation is key to their access to this species that has almost never been recorded as prey before (Dellinger & Trillmich, 1999; Páez‐Rosas & Aurioles‐Gamboa, 2010, 2014). Given the high burst swimming speed of scad (5.5 m/s measured in Atlantic mackerel, a similarly built species; Wardle & He, 1988), it would be nearly impossible for a single sea lion to catch scad as it would suffer from the confusion effect of the prey school and the difficulty of catching a fish swimming about as fast, and in bursts even faster, than a sea lion. Driving the fish school by joint manoeuvering and making it strand where fish can be picked up with little effort makes foraging for scad a cost‐efficient possibility. Earlier observations on sea lions hunting for sardines by Pierotti (1988) make it clear that attacking a bait ball jointly increases the hunting efficiency of sea lions enormously even when hunting fish who is a much slower swimmer (about 0.35 m/s) than scad. However, schooling scad never appear to group in the manner of a bait ball and would be inaccessible in open water for a single hunting sea lion.

Keeping the school together and controlling its swim direction requires a substantial degree of cooperative manoeuvering as otherwise the school could break up and escape. The likelihood of breakup of the school is evidenced by the observed escape of scad near the beach when the sea lions concentrated on the leading part of the school to drive it ashore, which sometimes allowed the posterior part of the school to break away and escape. This escape was made easier by the opportunistic local sea lions swimming in the opposite direction while approaching the core hunters from the shore, thereby disturbing their coordinated drive. In addition, the drive of the school was at times disrupted by the shadows of the pelicans flying ahead of the incoming sea lions which led to splitting up of the school and sometimes its escape. Both of these disturbances reduced the efficiency of the core hunters. This marked reduction in the effectiveness of the hunt in turn highlights that the long‐distance drive of the school must involve considerable cooperation of the core group. Simple synchronous action by hunters attracted to a rich resource would not achieve the directed move of the school. The effectiveness of the drive implies that the hunters perform complementary acts to keep the school on course to the target, both at the surface and during deeper dives.

It is plausible, although not possible to ascertain, that on days when hunts occurred in rapid succession, some of the local sea lions followed the active hunters and joined their efforts all the way from the source area near the outermost island. About 20 animals made up the local colony, a mixture of lactating females and their yearling pups, plus 2–3 subadult bulls taking turns patrolling the shallows (and actively joining the hunt). Most of the local females slept on the sand all day, not even waking when a hunt took place only meters away, usually departing singly in the late afternoon to feed and returning during the course of the following morning.

Our observations suggest, although without individual identification this cannot be proven, that there are a few individuals, apparently all females numbering a minimum of six, who have mastered the strategy of long‐distance herding of the prey, driving them intentionally toward a location where the geography can be used as a trap. Whereas some of the local animals cause disruption by approaching the fish school in a direction opposite to the hunt, at least part of the group of local sea lions appeared to contribute to the final drive into the cove. They may contribute to the overall success of the hunt by enclosing the fish at a time when almost invariably a part of the school manages to escape, as evidenced in the photographs. This is why we prefer to call them “opportunistic hunters” and avoid the term “scroungers” (Packer & Ruttan, 1988) most often used in the theoretical literature on foraging.

How and when the core hunting group of females decide to engage in a communal hunt remain unknown; however, their coordinated herding action suggests some planning. Whether such hunting groups involve cliques of animals (i.e., animals particularly strongly connected within the social network of a colony) as described by Wolf et al. (2007) remains unknown. At present, we have no information to infer how such planning is accomplished. The complementary action in driving the school to a target location may imply some sort of communication among the hunters, most likely visual, although underwater vocalizations cannot be ruled out. From our observations, we cannot conclude that there was any sort of specialization of roles among the core hunters. Sharing of the resource happened clearly in a rather chaotic manner and even involved other individuals and species, with absolutely no aggression displayed between them. Since the animals repeatedly hunted in this manner over years, the strategy must have been successful in terms of the energy intake of the hunters, but it certainly is not a strategy that is of general importance in terms of the overall energy intake at population level. It is well documented that most sea lions forage individually for pelagic or benthic prey (Jeglinski et al., 2012; Páez‐Rosas et al., 2017; Schwarz et al., 2021). It appears therefore highly likely that social learning is involved in the cooperative foraging we observed. A genetic basis of the cooperative foraging is unlikely. The standard for considering such a behavior as culture would include ruling out ecological and genetic influences and/or showing that the behavior does spread among more socially connected conspecifics. To resolve this issue, we need information on the individuals involved in cooperative hunting and on the original development of the cooperation.

Another case of cooperative hunting of Galapagos sea lions has recently been described by Páez‐Rosas et al. (2020). In the north of Isabela Island, sea lions drive tuna into narrow coves where the trapped tuna would strand themselves in an attempt to escape. The degree of cooperation achieved in the case of the yellowfin tuna hunt appears lower. In that situation, even single individual sea lions were sometimes observed to herd tuna into the trap successfully, where the panicked fish, much less maneuverable in shallow water than scad, stranded themselves in their attempted escape. The participation of several sea lions in these hunts could potentially be explained as primarily involving attraction of independent predators to a common resource, that is, mere similarity of action between individuals that happen to hunt in spatial proximity.

## 5 CONCLUSION

An important aspect of our observations lies in identifying the clear difference between “core hunters” whose strategy involves planning and collaboration by complementary behaviors to achieve their goal, and the “opportunistic hunters” who merely join a hunt organized and driven by others. It remains unclear to what degree the opportunistic hunters may contribute to hunting success or how often they reduce the successful outcome of the hunt and might therefore be called “scroungers”. Remarkably, the core hunters approach the area from afar. They must employ some sort of communication in order to start the hunt as a group which implies communication about a goal that is not present. As the local animals did not initiate these hunts independently, the hunting strategy appears to be a cultural trait. Until marking and/or telemetry can be applied to allow identification of individuals, it remains unclear where these animals come from and how they synchronize their planned hunting. Coordinated communal hunting appears all the more surprising given that sea lions usually hunt individually, and that communal hunting certainly has played no role in the evolution of their sociality.

## CONFLICT OF INTEREST

The authors have no conflict of interest to declare.

## AUTHOR CONTRIBUTION

**Tui De Roy:** Conceptualization (equal); Data curation (lead); Formal analysis (equal); Investigation (lead); Resources (lead); Validation (equal); Visualization (lead); Writing‐original draft (equal); Writing‐review & editing (equal). **Eduardo Ramon Espinoza:** Writing‐review & editing (supporting). **Fritz** **Trillmich:** Conceptualization (equal); Data curation (equal); Formal analysis (equal); Writing‐original draft (equal); Writing‐review & editing (equal).
